# The Role of Unsaturated Fatty Acid-Rich Dairy Products in Adipocyte Metabolism

**DOI:** 10.3390/molecules29235502

**Published:** 2024-11-21

**Authors:** Manuela Machado, Eduardo M. Costa, Sara Silva, Ana Maria Gomes, Manuela Pintado

**Affiliations:** Universidade Católica Portuguesa, CBQF Centro de Biotecnologia e Química Fina-Laboratório Associado, Escola Superior de Biotecnologia, Rua Diogo Botelho 1327, 4169-005 Porto, Portugal; mmachado@ucp.pt (M.M.); snsilva@ucp.pt (S.S.); amgomes@ucp.pt (A.M.G.); mpintado@ucp.pt (M.P.)

**Keywords:** fatty acid permeability, adipocyte metabolism, inflammatory cytokines, functional food

## Abstract

This study investigated the fatty acid profile, permeability, and metabolic effects of a functional yogurt enriched with pomegranate oil, focusing on its impact on lipid metabolism and inflammatory responses. The yogurt’s fatty acid composition was primarily composed of long-chain polyunsaturated fatty acids (54.37%), followed by saturated (29.34%) and monounsaturated fatty acids (16.36%). During in vitro digestion, a shift in fatty acid profile was observed, with a decrease in polyunsaturated and saturated fatty acids and a slight increase in monounsaturated fatty acids due to hydrolysis. This study further analyzed fatty acid permeability across Caco-2/HT29-MTX monolayers and 3T3-L1 cell uptake, revealing higher permeability for saturated fatty acids than unsaturated ones. In 3T3-L1 cells, permeated fatty acids induced higher lipolysis and increased adiponectin secretion without affecting leptin levels. Cytokine analysis indicated a decrease in pro-inflammatory markers, such as MCP-1, and a significant increase in anti-inflammatory cytokines like IL-10, suggesting potential benefits in reducing obesity-related inflammation. These results underscore the role of functional yogurts enriched with polyunsaturated fatty acids as promising agents for modulating lipid metabolism and inflammatory responses.

## 1. Introduction

Obesity is a chronic disease defined by excessive fat deposits that can impair health, leading to an increased risk of type 2 diabetes, heart diseases, and certain types of cancer. The most recent data from the World Health Organization (WHO) shows that one in eight people in the world live with obesity. Overweight and obesity are primarily caused by an imbalance between energy intake and energy expenditure, but some other causes can be associated, such as psychosocial and genetic factors. Beyond the health consequences, obesity is also linked to high global health expenditures. According to the WHO, the projected costs will reach USD 3 trillion annually by 2030 [1]. For these reasons, new prevention and management strategies are playing an increasingly important role. Diet has been widely recognized as playing a central role in obesity, so it is possible to reduce the risk of obesity through modifications to daily diet. In this way, the food industry has an important role in the development of new functional foods that can help in obesity management. Common dietary habits are characterized by the consumption of energy-dense foods, rich in sugar and saturated fat, which increases obesity [2]. On the other hand, a replacement of saturated fat with unsaturated fat is linked to a reduction in abnormal fat accumulation [3,4,5]. Among bioactive compounds, unsaturated fatty acids are of growing interest due to their important role in human health. Under physiological conditions, over 95% of dietary fat is stored in adipose tissue in the form of triglycerides (TGs) [6]. When energy expenditure increases, the stored TGs are hydrolyzed through lipolysis, releasing free fatty acids and glycerol. For this reason, lipolysis is one of the most important metabolic processes occurring in adipose tissue [6]. An imbalance between the supply of energy substrates and the energy requirements leads to the accumulation of their excess in adipocytes in the lipogenesis process.

Under normal conditions, adipose tissue stores excess energy as a result of hypertrophy (increase in cell size) and/or hyperplasia (increase in the number of cells). In obesity, hypertrophic adipocytes become resistant to the antilipolytic effect of insulin and reduce the capacity to accumulate lipids. Consequently, when adipocyte storage capacities are exceeded, fat accumulates in cells such as in the muscles and liver, leading to insulin resistance [7]. Some studies showed that unsaturated fatty acids, particularly long-chain polyunsaturated and conjugated fatty acids, possess a wide range of biological properties including antidiabetic and anti-obesity properties [8]. Regarding the anti-obesity effect, Vroegrijk et al. (2011) reported that mice fed high-fat diets with 1% pomegranate seed oil showed improved peripheral insulin sensitivity with no effect on liver insulin [9]. Moreover, commercially available supplements rich in punicic acid suppress lipid accumulation in 3T3-L1 cells. This effect was linked to a decrease in peroxisome proliferator-activated receptor gamma (PPAR-γ) expression levels [10]. Chou et al. (2012) also reported that punicic acid reduces 3T3-L1 adipocyte differentiation [11]. Using the same cellular model, Anusree et al. (2014) demonstrated that punicic acid (5–30 µM) increases adiponectin secretion and prevents reactive oxygen species generation [12]. Additionally, Hontecillas et al. (2009) reported that punicic acid activates both PPAR-γ and PPAR-α in white adipose tissue in mice. This activation was responsible for an improvement in glucose homeostasis and suppression of inflammation biomarkers [13]. Pomegranate seed oil was also used in a clinical trial with obese type 2 diabetic patients, with the results showing a reduction in IL-6, TNF-α, and fast blood sugar levels [14]. 

Taking this into consideration, this work aimed to evaluate the impact of a functional dairy product enriched with unsaturated long-chain fatty acids (LCFAs) in adipocyte metabolism. To achieve this, pre-digested yogurt samples were used, and intestinal permeability was assessed. Then, the ability of permeated compounds to influence adipocyte metabolism, including adipokine and cytokine production, was evaluated. 

## 2. Results and Discussion

### 2.1. Functional Yogurt Fatty Acid Profile

As can be seen in Table 1, the functional yogurt produced was mainly composed of long-chain polyunsaturated fatty acids (54.37%), saturated fatty acids (29.34%), and monounsaturated fatty acids (16.36%).

**Figure 1:**
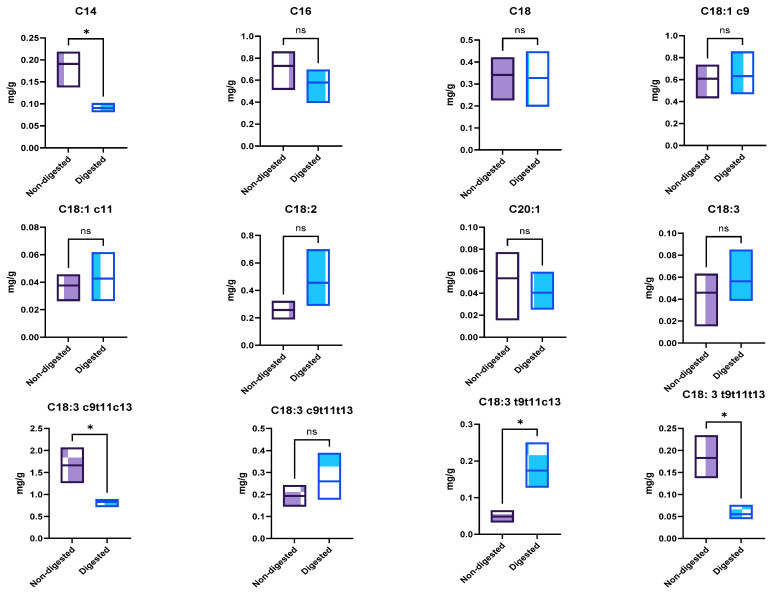
Effect of in vitro digestion on fatty acid quantitative profile. C14, myristic acid; C16, palmitic acid; C18, stearic acid; C18:1 *c*9, oleic acid; C18:1 *c*11, *cis*-vaccenic acid; C18: 2 *c*9*c*12, linoleic acid; C20:1, *cis*-gondoic acid; C18:3 *c*9*c*12*c*15, linolenic acid; C18:3 *c*9*t*11*c*13, punicic acid; C18:3 *c*9*t*11*t*13, α-eleostearic acid; C18:3 *t*9*t*11*c*13, catalpic acid; C18:3 *t*9*t*11*t*13, β-eleostearic acid; ns means no significant differences, and * means significant differences as determined by a one-way ANOVA test (*p* < 0.05).

This fatty acid profile was affected by gastrointestinal digestion, with the percentage of long-chain polyunsaturated fatty acids decreasing to 51.36% and the saturated fat to 20.20% and, oppositely, the monounsaturated fatty acids increasing to 28.12% probably due to the hydrolysis of unsaturated LCFAs. A more in-depth analysis (Figure 1) showed that the overall increase in monounsaturated fatty acids was not significant (*p* > 0.05); no significant differences were verified for oleic acid (C18:1 *c*9), *cis*-vaccenic acid (C18:1 *c*11), and *cis*-gondoic acid (C20:1). Concerning the saturated fatty acids, significant differences (*p* < 0.05) were only observed for myristic acid (C14). The polyunsaturated fatty acid fraction was more affected by digestion, with a significant (*p* < 0.05) reduction (53.93%) in conjugated fatty acids amount (C18:3 *c*9*t*11*t*13, α-eleostearic acid; C18:3 *t*9*t*11*c*13, catalpic acid; and C18:3 *t*9*t*11*t*13, β-eleostearic acid). The data reported in this work follow those reported in the literature [15,16,17].

### 2.2. Fatty Acid Permeability

**Figure 2:**
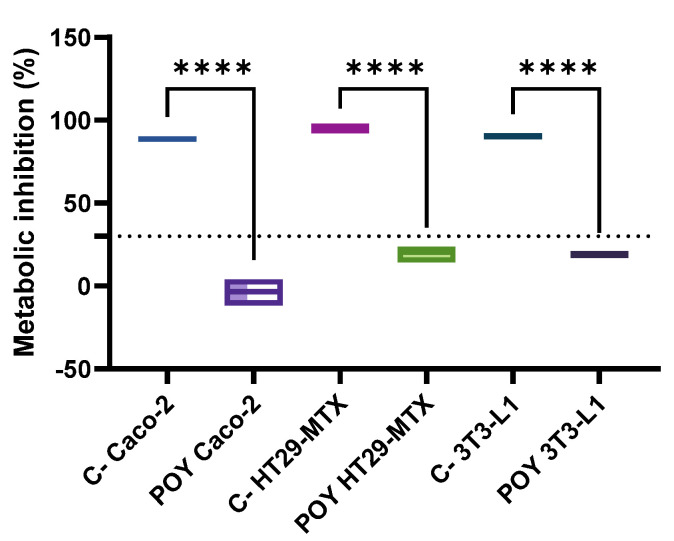
Pomegranate oil yogurt cytotoxicity toward the target cell lines. CT is the negative control (40% *v/v* DMSO). The dotted line represents the 30% cytotoxicity limit as defined by ISO 10993–5 [18]. **** means significant differences as determined by a one-way ANOVA test (*p* < 0.001).

The cytotoxicity of the POY samples was assessed. As shown in the Figure 2 below, an inhibitory effect on cellular metabolism across all tested cell lines was observed. Additionally, when analyzing the effect of the POY sample on Caco-2 cell metabolism, an apparent metabolic stimulation was observed, indicated by negative values for metabolic inhibition, suggesting a metabolic activity higher than that of the growth control.

The presence of digested yogurt in the apical compartments decreased by 14% the membrane TEER value compared to the control. This result is in accordance with those reported for permeability assays in Caco-2 monolayers and Caco-2/HT29-TMX co-cultures in the presence of fatty acids [19,20,21]. The reduction in TEER may indicate a loss of membrane integrity, which can be related to the amount of free fatty acids and their possible impact on cell membrane lipid bilayer, due to the increased paracellular absorption and consequently increased membrane fluidity [21,22]. Regarding the compound’s permeability (Table 2), the results showed that some fatty acids were not detected on the basolateral side; however, these compounds were retained by the Caco-2/HT29-MTX membrane and, in a few cases, were also metabolized by 3T3-L1 cells. In terms of permeability, saturated fatty acids showed higher apparent permeability than unsaturated fatty acids. Regarding the total amount of unsaturated LCFAs, it was observed that more than 25% was retained by the Caco-2/HT29-MTX membrane. In addition, small amounts of unsaturated fatty acids were uptaken by 3T3-L1 cells. A similar result has been reported for avocado oil-enriched dairy products [19]. Concerning the conjugated fatty acids, it is not possible to quantify them on the basolateral side, on the apical side, or in the 3T3-L1 cells; however, in the membrane, the presence of two conjugated linoleic acids was found. Some studies showed that conjugated linolenic acids, particularly, punicic, eleostearic and catalpic acids, can be metabolized by Caco-2 cells, resulting in conjugated linoleic acids (*t*9*t*11-CLA and *c*9*t*11-CLA). The mechanism of this conversion is not completely understood. Nevertheless, some authors suggest that the ∆13 double bonds in conjugated linoleic acids are reduced by an NADPH-dependent enzyme [23,24,25,26].

### 2.3. Effect on Adipocyte Metabolism

**Figure 3:**
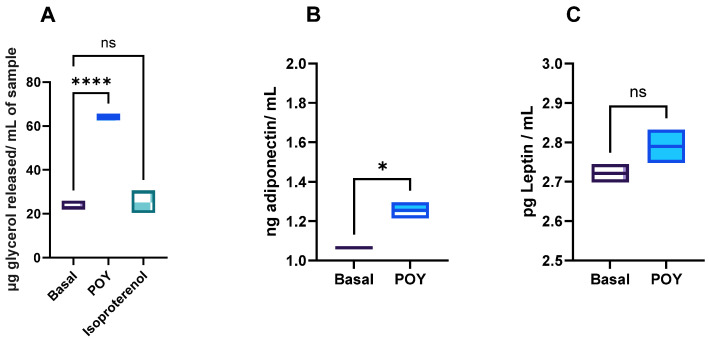
Effect of permeated pre-digested yogurt on lipolysis (**A**) and adipokine (adiponectin (**B**) and Leptin (**C**)) secretion in 3T3-L1 cells after 24 h of exposure. POY—pomegranate oil yogurt; ns means no significant differences, and * and **** means significant differences as determined by a one-way ANOVA test (*p* < 0.05 and *p* < 0.001).

The exposure of adipocytes to permeated fatty acids showed a higher rate of lipolysis compared to both basal activity and the assay control (isoproterenol). This effect may be attributed to the presence of unsaturated fatty acids in the permeated fraction, which can increase lipolysis and decrease triglyceride content in mature adipocytes [27]. The lipolytic effect of unsaturated fatty acids, particularly monounsaturated fatty acids (MUFAs), is linked to their ability to reduce PPAR-γ pathway activation [27,28]. Moreover, MUFAs may increase fatty acid oxidation rates and inhibit lipogenesis [29,30]. Data on adipokine secretion revealed a significant (*p* < 0.05) increase in adiponectin secretion compared to the basal condition (Figure 3). This result aligns with the lipolysis rate data, as adiponectin is inversely related to obesity; in other words, adiponectin levels rise as triglyceride storage decreases [27,31]. In contrast, leptin secretion is typically elevated in obesity, stimulating appetite and reducing sympathetic nerve activity [27,31]. No significant difference (*p* > 0.05) was observed in leptin secretion in this study (Figure 3). Studies using adipose tissue and cell models have shown that MUFAs can increase adiponectin secretion, consistent with our findings [32,33,34]. Additionally, a previous study on yogurt fortified with pomegranate oil as a source of long-chain unsaturated fatty acids showed an increase in adiponectin secretion [15]. However, while the presence of these fatty acids is generally associated with reduced leptin secretion, this effect was not observed in the present study.

**Figure 4:**
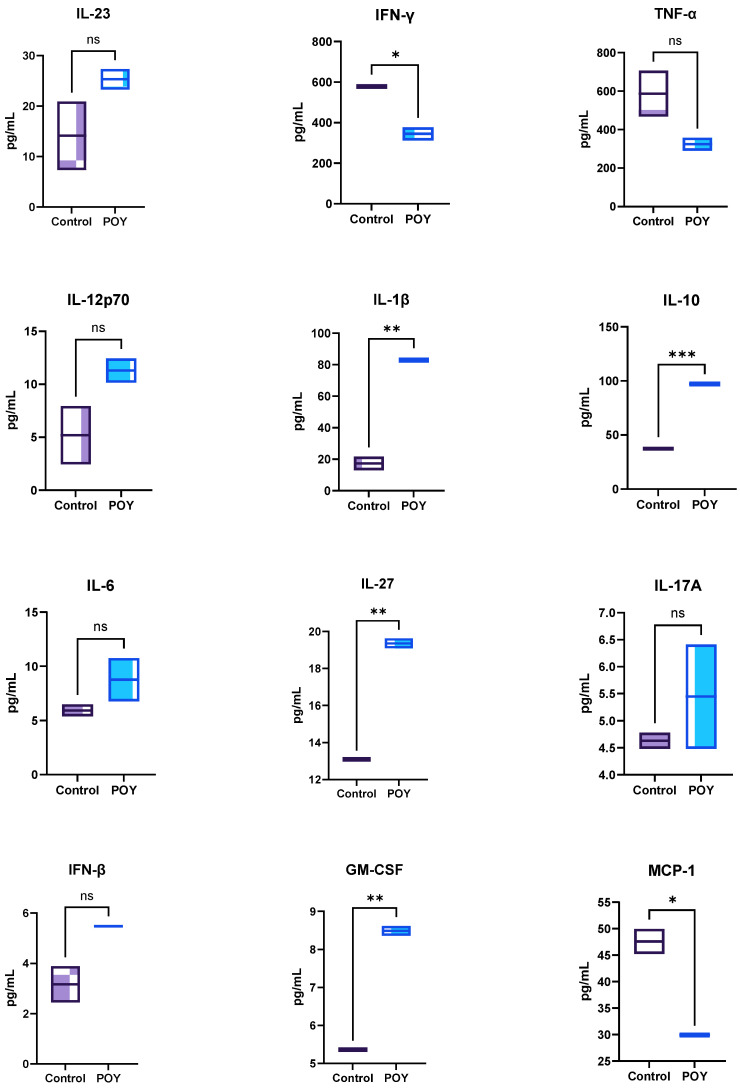
Cytokine production on the 3T3-L1 complex co-culture model basolateral side by the Caco-2/HT29-MTX membrane. POY—pomegranate oil yogurt; ns means no significant differences, and *, ** and *** means significant differences as determined by a one-way ANOVA test (*p* < 0.05, *p* < 0.01 and *p* < 0.001).

Adipose tissue expansion in obesity is associated with inflammatory changes within the tissue, contributing to a chronic, low-grade inflammatory state characterized by elevated levels of cytokines, chemokines, and acute-phase reactants. Previous studies have shown that dietary unsaturated fatty acids can modulate the inflammatory status of adipocytes [35,36,37]. As shown in Figure 4, significant differences (*p* < 0.05) were observed in the secretion of IFN-γ, IL-1β, IL-10, IL-17, GM-CSF, and MCP-1. Obesity is typically associated with high circulating levels of IL-6, TNF-α, and MCP-1 [31]. However, in the data reported in Figure 3, no significant differences (*p* > 0.05) were observed for these cytokines. Conversely, MCP-1 secretion significantly decreased (*p* < 0.05) compared to the basal activity, a change associated with weight loss [31]. Despite the high amount of saturated fatty acids in the permeated fraction, the presence of MUFAs, particularly oleic acid, may be responsible for this reduction, as some studies have shown MUFAs’ ability to inhibit MCP-1 secretion by blocking the NF-kB signaling pathway [32,33,38].

Regarding INF-γ, a significant (*p* < 0.05) decrease (ca 33%) was observed. INF-γ is a part of the group type II interferons which participate in innate and adaptative immune responses [39]. High levels of circulating IFN-γ are associated not only with the immune response of visceral adipose tissue but also with chronic inflammation and insulin resistance [39,40,41]. In the case of IL-1β, a significant increase (*p* < 0.05) was observed. The increase in IL-1β is linked to obesity and insulin resistance [39]. The increase in this cytokine is probably due to the presence of saturated fatty acids, particularly palmitic acid, which is recognized by their pro-inflammatory behavior [42]. In contrast with the previous cytokines, IL-10 has an anti-inflammatory role, and studies showed that IL-10 reduces diet-induced insulin resistance by reducing the response of macrophages. Moreover, IL-10 secretion increases the adipose thermogenesis [43]. In this work, the presence of permeated pre-digested fatty acids significantly (*p* < 0.05) increases (ca 63%) the IL-10 secretion. Concerning IL-27, a significant (*p* < 0.05) increase was observed in the POY samples. IL-27 is a heterodimeric cytokine included in the IL-12 family. The increase in their secretion is obesity-related and associated with insulin resistance and adipose tissue inflammation [44]. The increase in IL-27 reported in Figure 3 is corroborated by an animal study, which showed an increase in IL-27 serum levels after omega-3 supplementation [45]. Lastly, a significant (*p* < 0.05) increase in GM-CSF was observed. GM-CSF is a pro-inflammatory cytokine that has an important role in food intake reduction and weight loss [46]. Contrary to the data reported in this work, some studies showed a reduction in GM-CSF secretion after fish oil (rich in long-chain fatty acids) supplementation [47].

## 3. Materials and Methods

### 3.1. Yogurt Production, Digestion, and Fatty Acid Profile

Unsaturated LCFAs-rich yogurt was produced as described by Machado et al., 2023 [15]. Briefly, 1.5% (*w*/*w*) of pomegranate oil was added to semi-skimmed milk and pasteurized (85 °C, 5 min). Before fermentation, the samples were cooled to 45 °C and inoculated with natural yogurt. The resulting mixtures were incubated at 43 °C until pH reached 4.6. The final yogurts were cooled and stored at 4 °C. The in vitro gastrointestinal tract simulation was performed using the INFOGEST method, using 5 g of yogurt. After the intestinal phase, 1 mL aliquots were collected to determine the fatty acid profile, and the remaining sample was aliquoted and stored at −30 °C for further in vitro assays. The fatty acid profile was evaluated by gas chromatography after transesterification according to the method previously described by Machado et al. (2022) [16]. Fatty acid methyl esters were analyzed in a gas chromatograph Agilent 8860 (Agilent, Santa Clara, CA, USA), equipped with a flame ionization detector and a BPX70 capillary column (60 m × 0.25 mm × 0.25 μm; SGE Europe Ltd., Courtaboeuf, France). The analysis conditions were as follows: injector (split 25:1; injection volume 1 µL), injector and detector temperatures were 250 °C and 275 °C, respectively; hydrogen was used as a carrier gas at a flow rate of 1 mL/min. The oven temperature was initially at 60 °C and then increased to a final temperature of 225 °C. Supelco 37 (Sigma-Aldrich, Burlington, MA, USA) was used for the identification of fatty acids. Each sample was analyzed in triplicate.

### 3.2. Cellular Models

#### 3.2.1. Cell Lines

Three different cell lines were used in the current work: Human Caucasian colon carcinoma epithelial cells (CaCo-2, ECACC 86010202) and HT29-MTX E12 (ECACC 12040401) were acquired from the European Collection of Authenticated Cell Cultures. Mouse pre-adipocytes 3T3-L1 (ATCC CL-173) were acquired from American Type Culture Collection. Epithelial cells were cultured using Dulbeccos Modified Eagle’s Medium (DMEM; Gibco, Thermo Scientific, Waltham, MA, USA) supplemented with 10% (*v*/*v*) fetal bovine serum from Biowest (FBS; Nuaillé, France), 1% (*v*/*v*) of Penicillin-Streptomycin-Fungizone (Lonza Basel, Switzerland), and 1% (*v*/*v*) non-essential amino acids (Gibco, Thermo Scientific, MA, USA). Pre-adipocytes were cultured in DMEM with 10% (*v*/*v*) of calf bovine iron-fortified serum (ATCC, Manassas, VA, USA) and 1% (*v*/*v*) of Penicillin-Streptomycin-Fungizone. All cell lines were incubated at 37 °C under a humidified atmosphere comprising 5% CO_2_ and 95% air.

#### 3.2.2. Cytotoxicity Evaluation

Cytotoxicity evaluation was performed according to ISO 10993-5:2009, as described by Machado et al., 2023 [15]. Briefly, cells were seeded at 1 × 10^4^ cell/well in 96-well plates (Nunclon Delta, Thermo Scientific, Waltham, MA, USA). After 24 h, the culture media were carefully removed and replaced with culture media with digested POY samples (0.2 mg/mL of fatty acids). DMSO at 40% (*v*/*v*) in culture media was used as a death control, and a plain medium was used as a growth control. After 24 h, an MTT solution (0.5 mg/mL) was added to each well and incubated for 2 h. After this period, the content was removed and the MTT formazan crustal was dissolved with DMSO. Absorbance (570 nm) was measured using a microplate reader (Synergy H1, Biotek Instruments, Winooski, VT, USA).

#### 3.2.3. Co-Culture Models

Co-culture models were performed through adaptation of the methods previously described by [19]. Briefly, Caco-2/HT29-MTX co-cultures were seeded on the apical chamber of a 12-well Transwell (Corning, NY, USA) plate in a proportion of 90:10, respectively, to reach a monolayer with a final density of 1 × 10^5^ cells/cm^2^ in each insert and were then maintained for 21 d until assaying.

#### 3.2.4. Cell Monolayer Integrity

Membrane integrity was evaluated through transepithelial electrical resistance (TEER) using a Millicell ERS-2 Voltohmmeter (Merck, Darmstadt, Germany). Monolayers with TEER values between 150 and 250 cm^2^ were selected for permeability experiments.

#### 3.2.5. 3T3-L1 Metabolism Modulation

Pre-adipocytes were seeded (4 × 10^4^ cells/well) into 12-well plates and chemically differentiated according to ATCC guidelines as follows: when cells reached 100% confluence, the culture medium was replaced and the cells were incubated for 48 h. After that, the medium was replaced by a differentiation medium (90% of DMEM, 10% FBS, 1.0 µM Dexamethasone (G Biosciences, St. Louis, MO, USA), 0.5 mM 3-Isobutyl-1-methylxanthine (Sigma, Burlington, MA, USA), and 1 µg/mL insulin (Sigma, MO, USA)), and the cells were incubated for another 48 h. Then, the differentiation medium was replaced by adipocyte maintenance media (90% DMEM, 10% FBS, and 1 µg/mL of insulin). Every 2 d, the adipocyte maintenance medium was replaced until the cells reached more than 80% differentiation (12 d after induction).

After this period, 21-day-old Caco-2/HT29-MTX monolayers were transferred from the transwell plate and placed over the adipocyte’s monolayer, after which the media in the apical chamber were replaced with plain media (basal control), media with digested sample, or with DMSO at 10% (*v*/*v*) (stress control), and the plate was re-incubated for 6 h. Following incubation, the apical medium was collected, an aliquot from the basolateral side was collected, inserts were removed, and the plates were incubated for 18 h. Cell membranes were collected using 0.1 M of sodium hydroxide and stored at −80 °C until analysis. After 24 h, supernatants and adipocytes were collected and stored at −80 °C for further analysis. The fatty acid profile of apical and basal samples, cell membranes, and 3T3-L1 was determined as described in Section 3.1. The apparent permeability was calculated using the following equation:

$$Papp\left(cm/s\right)=\frac{dQ}{dt(A\times C0)}$$

where ***dQ*** is the total amount of permeated fatty acid (µg/mL), ***A*** is the diffusion area (cm^2^), ***C*0** is the initial concentration of fatty acids (µg/mL), and ***dt*** is the time of the experiment(s). The coefficient ***dQ***/***dt*** represents the flux of fatty acids across the monolayer. All assays were performed in duplicate.

Lipolysis was determined as previously described by Machado et al. (2022) [48]. Briefly, 25 µL of cell supernatants was mixed with 100 µL of glycerol-free reagent. The reaction mixture was incubated at room temperature for 15 min, and the absorbance was read at 540 nm in a microplate reader (Synergy H1, Biotek Instruments, Winooski, VT, USA). Adiponectin and leptin detection was performed with an enzyme-linked immunosorbent assay (ELISA) using Abcam’s Mouse Leptin ELISA kit and Mouse Adiponectin ELISA kit (Abcam, Cambridge, UK) according to the manufacturer’s instructions. The protein content of samples was determined using the BCA Pierce Assay Kit (Thermo Scientific, MA, USA) according to the manufacturer’s instructions. Leptin values were obtained in pg/mL in the sample and adiponectin in ng/mL. Each sample was analyzed in triplicate.

Immunomodulatory effects upon 3T3-L1 adipocytes present in the basolateral compartment were performed using a 13-analytes (IL-23, IL-1α, IFN-γ, TNF-α, CCL2 (MCP-1), IL-12p70, IL-1β, IL-10, IL-6, IL-27, IL-17A, IFN-β, and GM-CSF) mouse multiplex inflammation panel (LEGENDplex, Biolegend, San Diego, CA, USA) according to the manufacturer’s instructions. The results were obtained using a BD Accuri™ C6 flow cytometer gated according to the multiplex manufacturer’s instructions, and the results were expressed in pg/mL. Each sample was analyzed in quadruplicate.

### 3.3. Statistical Analysis

GraphPad Prism 10.0.0 (Boston, MA, USA) was used to carry out statistical analysis. All data were reported as the mean ± standard deviation. Shapiro–Wilk’s test was used to confirm the normality of data distribution. The results obtained were tested at a 0.05 significance level using a one-way ANOVA, followed by a multiple comparison test (Tukey) to identify statistically significant differences between samples.

## 4. Conclusions

The findings of this study highlight the functional yogurt’s unique fatty acid profile and its potential health benefits. The digestive process modified its fatty acid composition, specifically reducing polyunsaturated and saturated fatty acids while enhancing monounsaturated fatty acids. The permeability assays showed that saturated fatty acids were more readily absorbed across the Caco-2/HT29-MTX membrane, while unsaturated fatty acids exhibited limited permeability but were partially retained or metabolized. In adipocyte models, the permeated fraction enhanced lipolysis and adiponectin secretion, reflecting improved lipid metabolism. Additionally, the modulation of inflammatory cytokines, with decreased MCP-1 and increased IL-10 and GM-CSF, suggests that this functional yogurt could help counteract obesity-related inflammation. Overall, pomegranate oil-enriched yogurt offers promising metabolic and anti-inflammatory benefits, supporting its potential role in health-promoting dietary interventions.

## Figures and Tables

**Table 1:** Fatty acid profile of functional yogurt.

Fatty Acid	Amount (mg/g)
**C14**	0.22 ± 0.01
**C16**	0.84 ± 0.03
**C18**	0.40 ± 0.03
**C18:1 *c*9**	0.70 ± 0.1
**C18:1 *c*11**	0.04 ± 0.01
**C18:2 *c*9*c*12**	0.3 ± 0.1
**C20:1**	0.07 ± 0.01
**C18:3 *c*9*c*12*c*15**	0.06 ± 0.01
**C18:3 *c*9*t*11*c*13**	1.9 ± 0.3
**C18:3 *c*9*t*11*t*13**	0.22 ± 0.04
**C18:3 *t*9*t*11*c*13**	0.06 ± 0.01
**C18: 3 *t*9*t*11*t*13**	0.21 ± 0.04
**∑ Fatty acids**	5.0 ± 0.2

The results are expressed in mg/g and are the means of three determinations ± standard deviation. C14, myristic acid; C16, palmitic acid; C18, stearic acid; C18:1 *c*9, oleic acid; C18:1 *c*11, *cis*-vaccenic acid; C18: 2 *c*9*c*12, linoleic acid; C20:1, *cis*-gondoic acid; C18:3 *c*9*c*12*c*15, linolenic acid; C18:3 *c*9*t*11*c*13, punicic acid; C18:3 *c*9*t*11*t*13, α-eleostearic acid; C18:3 *t*9*t*11*c*13, catalpic acid; C18:3 *t*9*t*11*t*13, β-eleostearic acid.

**Table 2:** The fatty acid content in the apical and basolateral chamber, Caco-2/HT29-MTX monolayer, the compounds’ apparent permeability, and uptake by 3T3-L1 cells. nd—not detected.

	6 h					24 h
	Initial (µg/mL)	Apical (µg/mL)	Membrane (µg/mL)	Basolateral (µg/mL)	P_app_ (cm/s)	Cells (µg/mL)
**C14**	4.5 ± 0.3	nd	1.0 ± 0.1	nd	-	nd
**C16**	29 ± 6	5 ± 2	11 ± 5	6 ± 1	0.19 ± 0.02	1.3 ± 0.2
**C16:1**	nd	nd	3 ± 1	nd	-	3.6 ± 0.1
**C18**	16 ± 3	4 ± 1	5.7 ± 0.2	6.2 ± 0.2	0.4 ± 0.1	0.75 ± 0.03
**C18:1 *t*9**	nd	nd	5 ± 2	nd	-	nd
**C18:1 *c*9**	32 ± 8	6 ± 3	15 ± 7	5 ± 1	0.16 ± 0.04	5.4 ± 0.8
**C18:1 *c*11**	2.1 ± 0.4	nd	4 ± 3	nd	-	nd
**C18:2 *c*9*c*12**	23 ± 6	nd	3 ± 1	nd	-	nd
**C20**	nd	8 ± 5	5 ± 2	23 ± 1	-	11 ± 5
**C20:1**	2.02 ± 0.03	nd	1.1 ± 0.2	nd	-	nd
**C18:3 *c*9*c*12*c*15**	2.8 ± 0.3	nd	1.91 ± 0.03	nd	-	nd
**C18:2 *c*9*t*11**	nd	nd	2.93 ± 0.01	nd	-	nd
**C18:2 *t*9*t*11**	nd	nd	1.88 ± 0.02	nd	-	nd
**C20:3 *c*11*c*14*c*17**	nd	nd	nd	nd	-	2 ± 1
**C18:3 *c*9*t*11*c*13**	42 ± 6	nd	nd	nd	-	nd
**C18:3 *c*9*t*11*t*13**	13 ± 1	nd	nd	nd	-	nd
**C18:3 *t*9*t*11*c*13**	9 ± 1	nd	nd	nd	-	nd
**C18: 3 *t*9*t*11*t*13**	3 ± 1	nd	nd	nd	-	nd

The results are expressed as a mean ± standard deviation of three replicates. C14, myristic acid; C16, palmitic acid; C18, stearic acid; C18:1 *c*9, oleic acid; C18:1 *c*11, *cis-*vaccenic acid; C18: 2 *c*9*c*12, linoleic acid; C20, arachidic acid; C20:1, *cis*-gondoic acid; C18:3 *c*9*c*12*c*15, linolenic acid; C18:3 *c*9*t*11*c*13, punicic acid; C18:3 *c*9*t*11*t*13, α-eleostearic acid; C18:3 *t*9*t*11*c*13, catalpic acid; C18:3 *t*9*t*11*t*13, β-eleostearic acid.

## Data Availability

The data presented in this study are available from the corresponding author on request.
